# Lessons from Microglia Aging for the Link between Inflammatory Bone Disorders and Alzheimer's Disease

**DOI:** 10.1155/2015/471342

**Published:** 2015-05-19

**Authors:** Zhou Wu, Hiroshi Nakanishi

**Affiliations:** Department of Aging Science and Pharmacology, Faculty of Dental Science, Kyushu University, Fukuoka 812-8582, Japan

## Abstract

Bone is sensitive to overactive immune responses, which initiate the onset of inflammatory bone disorders, such as rheumatoid arthritis and periodontitis, resulting in a significant systemic inflammatory response. On the other hand, neuroinflammation is strongly implicated in Alzheimer's disease (AD), which can be enhanced by systemic inflammation, such as that due to cardiovascular disease and diabetes. There is growing clinical evidence supporting the concept that rheumatoid arthritis and periodontitis are positively linked to AD, suggesting that inflammatory bone disorders are risk factors for this condition. Recent studies have suggested that leptomeningeal cells play an important role in transducing systemic inflammatory signals to brain-resident microglia. More importantly, senescent-type, but not juvenile-type, microglia provoke neuroinflammation in response to systemic inflammation. Because the prevalence of rheumatoid arthritis and periodontitis increases with age, inflammatory bone disorders may be significant sources of covert systemic inflammation among elderly people. The present review article highlights our current understanding of the link between inflammatory bone disorders and AD with a special focus on microglia aging.

## 1. Introduction

Bone is a major site of immune responses, termed “osteoimmunology” [1]. An overactive immune response may initiate the onset of inflammatory bone disorders, such as rheumatoid arthritis (RA) and periodontitis, which can be significant sources of covert systemic inflammation. Because the prevalence of RA and periodontitis increases with age, it is important to recognize the contribution of these inflammatory bone disorders in the increasing elderly population worldwide. On the other hand, Alzheimer's disease (AD), the most common form of dementia, is known to be the most common cause of disability in elderly subjects. Although the molecular mechanisms involved in the etiology and pathogenesis of AD have not been completely elucidated, the accumulation of *β*-amyloid (A*β*) and hyperphosphorylation of tau in the brain is the hallmarks of AD, and microglia-mediated neuroinflammation is well known to be related to the onset and progression of AD due to the release of proinflammatory mediators [2, 3]. Interleukin-1*β* (IL-1*β*) is the key molecule involved in the neuroinflammation observed in cases of AD, as IL-1*β* drives the release of multiple inflammatory mediators by activated microglia, leading to a self-propagating cycle of neuroinflammation, which results in direct neurotoxicity and contributes to promoting the formation of dystrophic neurites [4].

It is well known that chronic systemic inflammation can alter the degree of neuroinflammation in the brain [5, 6]. RA and periodontitis, both chronic systemic inflammatory diseases, not only are associated with other systemic inflammatory diseases, such as atherosclerosis and diabetes, but also directly initiate or hasten the rate of progression of AD [7]. An increasing number of clinical studies have demonstrated the impact of RA and periodontitis on AD [8], and recent experimental studies have clarified the route of transduction of inflammatory signals from RA and periodontitis to the brain. We herein review the current understanding of the link between inflammatory bone disorders and AD.

## 2. Clinical Evidence for Inflammatory Bone Disorders as a Potential Risk Factor for AD

### 2.1. Rheumatoid Arthritis and AD

An inverse relationship between RA and AD has been reported since the early 1990s. A reduced prevalence of AD was described in RA patients who were long-term users of nonsteroidal anti-inflammatory agents (NSAIDs) analyzed in a postmortem survey [9], and a meta-analysis including 17 epidemiological studies demonstrated that NSAID use is a protective factor for AD onset [10]. Furthermore, a prospective study of 7,000 healthy subjects using NSAIDs for joint symptoms, including RA, showed that the long-term use of NSAIDs protects against AD [11]. Another systematic review of multiple prospective and nonprospective studies further showed that NSAID exposure is associated with a decreased risk of AD [12]. More recently, an increased risk of cognitive impairment in patients with midlife RA was confirmed based on a 21-year follow-up of the association between RA or arthritis and dementia/AD in several case-control and hospital- and register-based studies, which indicates that the presence of joint disorders, especially RA, in midlife appears to be associated with a worse cognitive status later in life [13].

### 2.2. Periodontitis and AD

The first hypothesis of a positive link between periodontitis and AD was raised in 2008. Kamer et al. proposed that periodontitis induces systemic inflammation, which stimulates the production of A*β* and tau protein in the brain, leading to Alzheimer's neuropathology [14]. In addition to the effects of low-grade chronic inflammation itself, periodontitis causes or promotes other chronic systemic inflammatory diseases, including atherosclerosis, cardiovascular disease, and diabetes, indicating that periodontitis is a significant source of systemic inflammatory molecules [15]. Based on the contribution of periodontitis to systemic inflammation, and the potential role of systemic inflammation in the onset of neuroinflammation, it is reasonable to consider that chronic periodontitis is a risk factor for the incidence and progression of AD.

There is growing clinical evidence that chronic periodontitis is closely linked to the initiation and progression of AD. Noble et al. identified a cross-sectional association between a serologic marker of a common periodontitis pathogen,* Porphyromonas gingivalis* (*P. gingivalis*), and poor cognitive test performance among patients older than 60 years in the Third National Health and Nutrition Examination Survey (NHANES-III) [16]. This preliminary study suggested that periodontitis is associated with cognitive impairment, especially in the elderly. Furthermore, Stein et al. examined the serum levels of antibodies against periodontal bacteria in participants who were eventually diagnosed with AD in comparison with that observed in non-AD controls [17] and found elevated levels of antibodies to periodontal bacteria years before the onset of cognitive impairment, suggesting that periodontitis potentially carries a risk of AD development and/or progression.

More recently, Poole et al. assessed the presence of the major three periodontal bacteria, the so-called “red complex” including* Treponema denticola*,* Tannerella forsythia,* and* P. gingivalis* and/or bacterial components in the brain tissue of individuals with and without dementia [18]. The authors obtained statistically significant evidence of the presence of lipopolysaccharide (LPS) from* P. gingivalis* in the AD cases, thus confirming that LPS from periodontal bacteria can access the AD brain during life. Moreover, Riviere et al. detected oral* Treponema* in the trigeminal ganglia, brain stem, and cortex and hippocampus of AD patients using molecular and immunological techniques [19].

**Figure 1:**
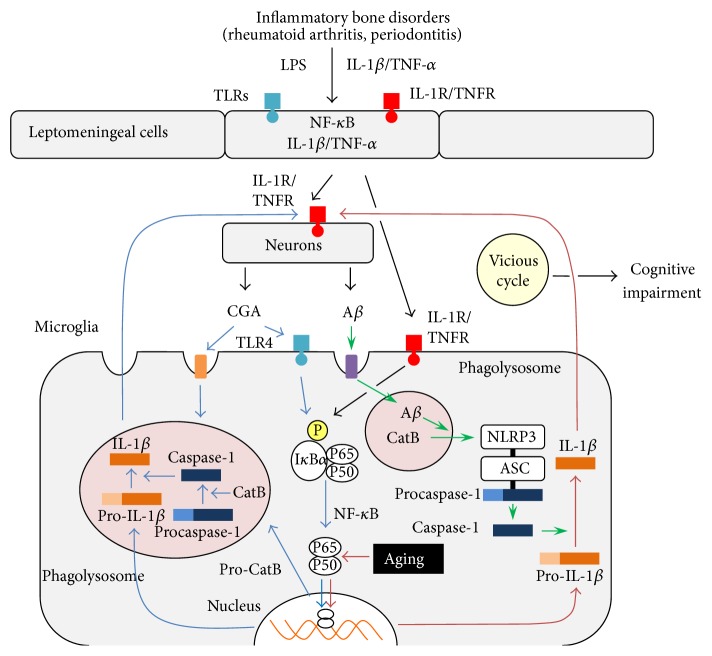
A schematic illustration of the transduction of signals from inflammatory bone diseases to brain-resident microglia through the leptomeninges and two different pathways for the IL-1*β* production activated by CGA and A*β* in microglia. In individuals with inflammatory bone diseases, IL-1*β* and TNF-*α* secreted by macrophages and periodontal bacterial components, including LPS, activate IL-1R/TNFR and TLRs localized on the surface of leptomeningeal cells to secrete IL-1*β* and TNF-*α*. IL-1*β* and TNF-*α* then stimulate both brain-resident microglia and neurons. After stimulation, the neuronal production and secretion of CGA and A*β* is increased. CGA and A*β* subsequently activate two different pathways for the IL-1*β* production in microglia, the NLRP3 inflammasome-CatB pathway via A*β* (open arrows), and the phagosome-CatB pathway via CGA (blue arrows). The NF-*κ*B pathway activated during aging (red arrows) supports the production and secretion of IL-1*β* by A*β*. The leptomeningeal cell-neuron-microglia interactions form a vicious cycle of IL-1*β* production, culminating in the onset of cognitive impairment in AD.

Taken together, clinical evidence suggests that the chronic inflammation associated with inflammatory bone disorders may have an important role in increasing the risk of cognitive impairment in cases of AD (Figure 1). However, the exact route by which these disorders transduce systemic inflammatory signals into the brain remains unclear.

## 3. Oxidative Damages and Cellular Stress Responses in the Inflammatory Bone Disorders and AD

### 3.1. Oxidative Damage in Inflammatory Bone Disorders

Receptor activator of nuclear factor-*κ*B ligand (RANKL) is a critical factor for the pathogenesis of inflammatory bone disorders due to its requirement for both the formation and activation of osteoclasts [20, 21]. For this reason, inhibition of the RANKL expression represents an innovative therapeutic target for controlling osteoclast formation in inflammatory bone disorders [20]. The nuclear factor-*κ*B (NF-*κ*B) pathway is central for regulating the RANKL-dependent osteoclast formation, function, and survival [22–24], which explains the inhibitory effects on osteoclast formation induced by the prevention of NF-*κ*B activation [22, 25]. Furthermore, reactive oxygen species (ROS) act as intracellular signaling molecules involved not only in the regulation of RANKL-dependent osteoclast formation but also in the peroxidation of lipids and oxidative damage to proteins and DNA [26, 27] as well as RANKL in turn to increase the ROS levels. It has been identified that a decrease in the ROS levels results in a reduction in osteoclast formation and attenuation of bone destruction, as found in both* in vitro* and* in vivo* models [27]. This raises the possibility that antioxidants may be therapeutic targets for the treatment of inflammatory bone disorders.

### 3.2. Oxidative Damage in AD

Oxidative stress-induced cell damage is the major component of harmful cascades activated in the development of aging-related neurodegenerative disorders, including AD [28–30]. Numerous reports have provided the direct morphological and biochemical evidence indicating a connection between oxidative stress and cell death in the brain of patients with AD [31–33]. Furthermore, ROS accumulate unfolded or misfolded proteins in the AD brain [34], and increasing evidence supports the role of ROS in the pathogenesis of AD, as A*β* oligomers directly generate hydrogen peroxide [35]. Moreover, the overproduction of ROS under condition of oxidative stress acts as a second messenger in signal transduction cascades leading to the activation of mitogen-activated protein kinases (MAPK), as the intracellular redox state of cells regulates cellular signaling pathways [36, 37]. The major MAPK subfamilies, c-Jun N-terminal kinase, p38, and extracellular signal-regulated kinase 1/2 are known to be cell death factors produced in response to oxidative stress [38, 39]. Indeed, the levels of phosphorylated MAPKs are increased in the postmortem brains of AD patients [40–42]. Therefore, antioxidant therapy is considered to be a promising approach for the prevention and clinical management of AD [43, 44].

### 3.3. Redox-Dependent Control of Oxidative Damage and Cellular Stress Responses: Relevance to Antioxidant Strategies

The modulation of endogenous cellular defense mechanisms represents an innovative approach to providing therapeutic interventions for diseases causing chronic tissue damage, including inflammatory bone disorders as well as AD. Cellular stress response consists of prosurvival pathways controlled by cytoprotective genes called vitagenes [45] that stimulate the production of molecules endowed with antioxidant and antiapoptotic potential [46]. Vitagenes include members of the heat shock protein (Hsp) family, such as heme oxygenase-1 and Hsp72, sirtuins, and the thioredoxin/thioredoxin reductase system [47, 48]. Increasing evidence suggests that the Hsps promotes cytoprotective conditions in the human disease states, such as chronic inflammation, bone diseases, and AD, as thoroughly reviewed by Calabrese et al. [46–48]. Furthermore, previous studies have indicated that sirtuin 1 is a potent inhibitor of NF-*κ*B transcription [49]. Therefore, vitagenes are emerging candidates as pharmacological agents for treating antioxidative damages [50].

## 4. Inflammatory Bone Disorders and Neuroinflammation

### 4.1. IL-1*β* Processing in Inflammatory Bone Disorders

Bone is sensitive to an overactive immune responses, which are mediated by macrophages, dendritic cells, and T cells, for diverting proinflammatory mediators in the bone microenvironment. In particular, the dramatic impact of proinflammatory mediators on bone cells, including osteoclasts and osteoblasts, is a key component of the pathogenesis of inflammatory bone disorders.

IL-1*β* is considered to be a critical inducer of the pathogenesis and tissue damage observed in cases of inflammatory bone disorders, including RA [51] and periodontitis [52], as IL-1*β* impairs the migration of osteoblasts [53] and upregulates the RANKL expression induced by osteocytes [54]. Our recent studies suggest that osteoclast precursors are capable of producing multiple mediators [20]. Furthermore, IL-1*β* is used as a biomarker to assess the therapeutic outcomes of patients with chronic periodontitis [55]. Therefore, IL-1*β* has become a focus of research due its potential as an attractive therapeutic target for inflammatory bone disorders [20].

The processing and secretion of IL-1*β* are tightly regulated by a two-step mechanism. The first step consists of the transcription and production of pro-IL-1*β* which depends on the activation of NF-*κ*B by Toll-like receptors (TLRs), and the second step consists of the activation of inflammasomes, which in turn activate procaspase-1. Inflammasomes are multiprotein oligomers consisting of the NOD-like receptor (NLR) family, including the pyrin domain-containing 3 (NLRP3), and the non-NLR family proteins, melanoma 2 receptor, and procaspase-1. Upon activation, the NLRP3 inflammasome binds to an adaptor protein, ASC, which in turn recruits procaspase-1 for autoactivation. Finally, caspase-1 cleaves pro-IL-1*β*, which is then secreted in a mature form [56]. Recent studies have provided evidence that the NLRP3 inflammasome is involved in the pathogenesis of both RA and chronic periodontitis. For example, Walle et al. showed that the negative regulation of NLRP3 inflammasome activation induced by A20 markedly protects against the onset of RA-associated inflammation and cartilage destruction, highlighting the contribution of the NLRP3 inflammasome to the pathology of RA [57]. Similarly, Park et al. found significantly higher levels of the NLRP3 inflammasome and caspase-1 in the gingival tissues of patients with chronic periodontitis compared to that observed in healthy controls. Furthermore, the activation of both the NLRP3 and AIM2 inflammasomes is necessary for IL-1*β* secretion after stimulation with* P. gingivalis*, the main bacteria that induces periodontitis [58].

### 4.2. Peripheral Fiber Sprouting in Inflammatory Bone Disorders

Peripheral nervous fiber sprouting is observed in inflammatory bone disorders and considered to be associated with pain, the major symptom of inflammatory bone disorders. However, our previous studies using an animal model of RA, adjuvant arthritic (AA) rats, showed that sprouting of peripheral sensory fibers is closely linked with bone remodeling and local inflammatory processes [59]. Infiltrated macrophages and CD4^+^ T cells produce nerve growth factor (NGF) and express its high-affinity receptor, TrkA, in the inflamed synovial tissues. However, a dense network of its low-affinity receptor, p75-positive nerve fibers, with numerous terminal varicosity, is also observed. These findings suggest that infiltrating mononuclear cells secrete NGF in an autocrine or paracrine manner in the inflamed synovium to promote sensory nerve fiber sprouting, as most of new sprouting GAP-43-positive fibers are calcitonin gene related peptide (CGRP)-positive sensory fibers located around osteophytes. However, the surgical reduction of CGRP-positive sensory fibers via resection of the sciatic nerves prior to adjuvant injection suppresses the size of osteophytes and delays the recovery of inflammation due to the effects of prolonged infiltration of Th1 cells in the synovial tissues in AA rats. These observations suggest the involvement of sporting sensory fibers in the bone remodeling and local inflammatory processes observed in inflammatory bone disorders.

More recently, the function of sprouting sympathetic fibers in inflammatory bone disorders has also been noted. Longo et al. showed that the sprouting of sympathetic fibers into the upper dermis of the skin, which results from increases in the mature NGF levels in the skin following joint and bone damage, is present in AA rats. The pharmacological suppression of the sympathetic fiber function with systemic guanethidine significantly decreases the pain-related behavior associated with arthritis in these animals, thus suggesting that sprouting sympathetic fibers contribute to the pain-related behavior associated with inflammatory bone disorders [60]. Furthermore, the dense sympathetic innervation of joints suggests the importance of this phenomenon in the onset of inflammatory bone disorders, as the sympathetic system controls the blood flow in the joint as well as the degree of vascular permeability and thus influences the extent of inflammation, as immune cells exhibit adrenergic signaling pathways. Therefore, the sprouting of sympathetic fibers may have local effects on osseous tissues in addition to effects on the local and systemic immune functions in cases of inflammatory bone disorders [61]. Taken together, these findings indicate that inflammatory bone disorders are a source of chronic systemic inflammation.

### 4.3. Inflammatory Bone Disorders and Neuroinflammation

It is well known that chronic systemic inflammation has causal links to neuroinflammation via the actons of systemically released proinflammatory mediators including IL-1*β* and that microglia are the primary brain cells responding to systemic inflammation [5, 6]. Repeated LPS-induced chronic systemic inflammation in mice results in prolonged IL-1*β* production as well as microglial activation in the brain [62]. Furthermore, mice subjected to systemic inflammatory challenges in late gestation are predisposed to develop AD-like neuropathology during the course of aging. These mice also display chronic elevation of IL-1*β*, an increased expression of hippocampal amyloid precursor protein and its proteolytic fragments and enhanced tau phosphorylation, thus resulting in a significant impairment of working memory in old age. More importantly, this phenotype is strongly exacerbated when the prenatal infection is followed by a second systemic inflammatory challenge in adulthood, further suggesting that systemic inflammation represents a major risk factor for the development of AD [63].

Several routes by which systemic immune signals may be transmitted to the brain have been studied intensively [5, 6]. First, the direct pathway involves the circumventricular organs, specialized regions lacking a contiguous blood-brain barrier. In circumventricular organs, pathogen-associated molecular patterns induce the production and release of proinflammatory mediators stimulated by macrophage-like cells expressing TLRs. These cytokines are able to enter the brain by volume diffusion. A second route involves the IL-1 receptors located on the perivascular macrophages and endothelial cells of brain capillaries. The activation of IL-1 receptors by circulating mediators initiates the release of cytokines into the brain, without the physical entry of constituents across the blood-brain barrier. A third route comprises the overflow of cytokine transporters into the systemic circulation, which then allows cytokines to gain access to the brain through these transport systems. A fourth route involves the transmission of systemic immune signals via the autonomic nervous system. Systemic cytokines directly stimulate primary afferent nerves, such as the vagus nerve, which in turn activate central pathways involved in sickness behavior.

In addition to these four “classical” routes, we further found that a leptomeningeal pathway may be involved. The leptomeninges covering the surface of the brain parenchyma provide a physical boundary at the cerebrospinal fluid-blood barrier. The activation of leptomeningeal cells by circulating cytokines induces the production and release of proinflammatory cytokines into the brain [64, 65]. Therefore, leptomeningeal cells are able to transmit signals from systemic immune cells into the brain-resident microglia (Figure 1).

## 5. Age-Dependent Differences in the Microglial Responses Acting via Leptomeninges in Inflammatory Bone Disorders

### 5.1. Age-Dependent Differential Microglial Responses in AA

It is interesting to note that inflammatory bone disorders induce age-dependent differential responses in microglia. Using a model of RA, rat AA, we found that activated microglia in the proximity of the leptomeninges produce anti-inflammatory cytokines, such as IL-10 and transforming growth factor-*β*1 (TGF-*β*1), in young adult AA rats [6, 66, 67]. In contrast, activated microglia in close proximity to the leptomeninges in middle-aged AA rats produce IL-1*β* and exhibited an increased expression of prostaglandin E_2_ (PGE_2_) synthesizing enzymes, such as cyclooxygenase-2 and microsomal prostaglandin synthase-1 [68]. In cultured leptomeningeal cells, IL-1*β* and PGE_2_, respectively, caused the marked loss of occludin and ZO-1, two major tight junction proteins: pretreatment with IL-10 and TGF-*β*1 significantly antagonizes these effects. Therefore, chronic systemic inflammation induces age-dependent phenotypic changes in microglia, yielding an anti-inflammatory cell phenotype in young rats and a proinflammatory cell phenotype in middle-aged rats. Furthermore, age strongly influences the barrier functions of the leptomeninges as a result of the age-dependent differential microglial responses in the setting of inflammatory bone disorders [68].

### 5.2. Functional Outcomes of Differential Microglial Phenotypic Changes in AA

These observations prompted further investigation of the functional outcomes of the resultant differential microglial phenotypic changes noted under condition of chronic systemic inflammation. We therefore examined the effects of chronic systemic inflammation on long-term potentiation (LTP) in the hippocampus in young adult and middle-aged rats, as LTP is the cellular substrate for learning and memory [69]. Consequently, the incidence of LTP in the Schaffer collateral-CA1 synapses was not affected in the young adult AA rats, whereas the formation of hippocampal LTP was significantly impaired in the middle-aged AA rats. The systemic administration of minocycline, a known inhibitor of microglial activation, significantly restored the magnitude of LTP in middle-aged AA rats. These observations suggest that chronic systemic inflammation induces deficits in the hippocampal LTP in middle-aged rats via the effects of neuroinflammation, which is primarily induced by brain-resident microglia.

Therefore, it is considered that microglia may be primed during aging, even by middle age. Primed microglia are hyperresponsive to secondary stimuli and can thus produce an exaggerated inflammatory response in the brain. It is also considered that age-dependent autophagic and lysosomal dysfunction allows for the dominance of ROS-hypergenerating older mitochondria in microglia. The increased levels of intracellular ROS, in turn, activate redox-sensitive transcription factors, such as NF-*κ*B, to provoke an exaggerated inflammatory response [70]. Therefore, increased oxidative stress and the resultant activation of redox-sensitive transcription factors observed during aging may drive the emergence of senescent-type microglia (microglia aging). This may explain why A*β*, which is not capable of sufficiently activating NF-*κ*B, is able to induce the secretion of IL-1*β* by activated microglia isolated from the aged mouse brain but cannot induce IL-1*β* secretion from the young adult mouse brain [71]. These observations may partly explain why senescence is an important causative factor for AD. It is important to note that the inflammatory bone disorders RA and periodontitis are generally found in the middle-aged and older populations, thus further indicting the risk association between inflammatory bone disorders and AD.

## 6. A***β*** and Chromogranin A (CGA): Key Players in the Neuroinflammation in the AD Brain

### 6.1. Activation of the NLRP3 Inflammasome-Cathepsin B (CatB) Pathway by A*β*

Latz's group has proposed a model for the activation of the NLR family, NLRP3 inflammasomes. According to this model, the phagocytosis of various molecules, including fibrillar A*β*42 and silica crystals, by LPS-primed microglia/macrophages causes phagosomal destabilization and lysosomal rupture. The subsequent secretion of CatB into the cytoplasm triggers the activation of the NLRP3 inflammasome directly or indirectly, thereby leading to the production and secretion of mature IL-1*β* [66, 67]. Recently, CatB was found to directly interact with the leucine-rich-repeat domain of NLRP3 [72]. After activation, the NLRP3 inflammasome mediates procaspase-1 activation to promote the processing and secretion of IL-1*β*. Therefore, caspase-1 is an essential enzyme for IL-1*β* production, as it is required for the processing of inactive precursors into mature, active forms that can then be secreted from cells (Figure 1). Latz's group also recently reported that NLRP3-deficient mice carrying mutations associated with familial AD show improvements in spatial memory deficits, reductions in the expression of caspase-1 and IL-1*β* in the brain, and enhanced A*β* clearance [73]. These observations suggest that A*β* activates microglia surrounding plaques to induce IL-1*β* production via the NLRP3-CatB pathway.

### 6.2. Activation of the Phagolysosome-CatB Pathway by CGA

In addition to A*β*, CGA, a neurosecretory acidic glycoprotein, is found in 30–40% of AD neuritic plaques [74], and CGA-positive plaques are surrounded more frequently by hyperactivated microglia in comparison to that observed in the case of A*β*-positive neuritic plaques [72]. More importantly, CGA alone is capable of inducing IL-1*β* production by microglia, whereas A*β* induces the IL-1*β* production only by LPS-primed or senescent-type microglia [71].

There is evidence suggesting that CatB is associated with the maturation of pro-IL-1*β* in the endosomal/lysosomal system, as CatB can effectively cleave procaspase-1 in a cell-free system only at an acidic pH [75, 76]. We recently demonstrated that CatB and caspase-1 are colocalized and that CA-074Me markedly inhibits the caspase-1 expression in the CGA-induced proteolytic processing of procaspase-1 to its mature form in the lysosome-related vesicles of microglia, which contain inactive forms of IL-1*β* [71, 77]. Furthermore, there are no signs of any leakage of CatB in microglia following treatment with CGA. The typical size of primary lysosomes is less than 1 *μ*m in diameter, whereas the mean diameter of CatB-containing enlarged lysosomes in CGA-stimulated microglia is 4.2 *μ*m. These findings are consistent with previous observations showing that IL-1*β* and CatB are colocalized in phagolysosomes and that the secretion of IL-1*β* involves the exocytosis of phagolysosomes in LPS-activated human monocytes [77, 78] (Figure 1). However, the possibility that CatB is indirectly involved in the activation of caspase-1 via the proteolytic maturation of caspase-11 cannot be totally ruled out, as caspase-11 can activate procaspase-1 [79]. Furthermore, CGA leaked from damaged neurons also activates microglia surrounding plaque to induce IL-1*β* production at an earlier age than fibrillar A*β* via the phagolysosome-CatB pathway [71].

### 6.3. Increase in A*β* and CGA following the Onset of Inflammatory Bone Disorders

Our preliminary experiments showed that AA and chronic systemic treatment with* P. gingivalis* LPS induces the intracellular accumulation of A*β* and CGA in hippocampal pyramidal neurons, leading to memory impairment in middle-aged animals (Wu et al., unpublished observations, Figure 1). However, further studies are needed to clarify the mechanism underlying the accumulation of A*β* and CGA in presence of inflammatory bone disorders. It is noteworthy that the IL-1*β* secreted from activated microglia further accelerates tangle formation in cortical neurons via tau hyperphosphorylation, thus indicating that activated microglia may also play important roles in tau pathology in AD [80].

## 7. Conclusion

Recent evidence indicates that inflammatory bone disorders are potential risk factors for AD. In individuals with chronic inflammatory bone disorders, proinflammatory blood cells and bacterial components, including LPS, activate the receptors localized on the surface of leptomeningeal cells, which in turn activate brain-resident microglia to evoke neuroinflammation (Figure 1). The intense neuroinflammation evoked by senescent-type microglia may contribute to the initiation and progression of AD, resulting in cognitive impairment. Therefore, providing early treatment of inflammatory bone disorders may delay the onset and limit the severity and/or progression of AD. More importantly, microglia undergo several morphological and functional changes involving the induction of exaggerated neuroinflammation in response to systemic inflammation during normal aging. Therefore, pharmacological approaches aimed at rejuvenating senescent-type microglia may also constitute a promising avenue for future research to reduce the risk of AD.
